# Remarks on Vaccine Inoculation

**Published:** 1801-12

**Authors:** John Ring

**Affiliations:** New Street, Hanover Square


### *Remarks on Vaccine Inoculation, by* Mr. Ring, *Member of the Royal College of Surgeons in London.*

To the Editors of the Medical and Physical Journal.

Gentlemen,

In your Journal for October is an article, ſtating, that the Cow-pox appeared in the ſixth century, proving extremely fatal; fatal; and that the Small-pox in the human ſubject originated from that ſource. Theſe errors are ſo palpable, that I ſhould not have expected much miſchief to flow from them, had they not been copied into another publication, which may give them the greater ſanction. It is certain that the diſeaſe to which the Biſhop of Lauſanne alludes, is the murrain; and it is equally certain, that the cow can neither receive, nor communicate, the Small-pox.

The report, that Vaccine Inoculation had ſucceeded in Ruſſia, proves to have been premature; we have, however, great reaſon to believe, ſucceſs in that quarter will not long be wanting.

I lately received a letter from a medical practitioner in the county of Berks, in which he aſks, Whether the maſs of evidence in favour of Vaccine Inoculation is ſo great as it is preſumed to be? This query was occaſioned by ſome *pretended* failures; for it is not likely, that two or more inſtances of real failures ſhould happen at one time, and in the practice of one profeſſional man; when ſo many other individuals have inoculated from a hundred to a thouſand each, all of whom have reſiſted every form of the Variolous infection, to which they have been expoſed.

Excluſive of other fallacious appearances, which have given riſe to miſtakes in the practice of the new inoculation, ſuch as anomalous puſtules, there is another fertile ſource of error. The chicken-pox is frequently miſtaken for the Small-pox. Several inſtances of this kind have fallen under my own obſervation within a ſhort time. Hence, perſons who had undergone Vaccine Inoculation, have been ſuppoſed to have the Small-pox in the natural way, when the diſeaſe was the Chicken pox; and this latter has in many caſes been inſerted by way of inoculation, inſtead of the Small-pox.

While unfavourable caſes are circulated with great induſtry, by profeſſed enemies of Vaccine Inoculation, or its *pretended friends*, it appears to me, that the real friends of the practice, lulled into a ſtate of falſe ſecurity, are become rather remiſs; and, being convinced themſelves, imagine that the public are equally convinced. Hence they leave the field to their more active opponents, who improve this favourable occaſion to their own advantage.

The mildneſs of the Cow-pox is now undeniable; but, in regard to its efficacy as a preventive, there is a doubt remaining in the minds of many perſons, not much experienced in the practice, which challenges farther inveſtigation. I conſider it, therefore, as a duty to the public, to the cauſe of truth, and to Dr. Jenner, to declare, that having inoculated with Vaccine virus as many as my other avocations would permit, and having particularly fought for patients in thofe places, where the Small-pox raged, I have never yet been able to difcover a fingle inftance, where any one who had undergone Vaccine Inoculation proved fufceptible of the infection of the Small-pox.

Thefe proceedings have been watched with a jealous eye, by perfons not interefted in concealing any real or apparent failure; whofe filence, after the moft fevere fcrutiny, is a ftronger argument in favour of the new practice, than any atteftation of mine. I have now inoculated above eleven hundred, who have gone through the Vaccine affection in a regular way; and, after convincing myfelf of the certainty of this preventive, by repeated inoculations with Variolous matter, left the tafk of putting the remaining part of my patients to the teft of Variolous contagion in the natural way, to their parents; and their compliance with my requeft has been fo general, that, independant of accidental and unavoidable expofure, I have reafon to believe at leaft nine out of ten have been voluntarily fubjected to that teft.

It neceffarily follows from thefe obfervations, that the preventive employed by fome practitioners is lefs efficacious; or the teft to which their patients are afterwards fubmitted, more powerful. In order to decide this queftion, if any gentleman who has infected two, ten, or twenty of his own Vaccine patients with Variolous matter, will take the trouble of inoculating mine with the fame, I will furnifh him with at leaft a thoufand, on whom he may exercife his fkill. This offer the opponents of Vaccine Inoculation, and its *pretended friends* ought to accept; or, in decency, to be filent.

One remark more is loudly called for. Your Journal, as well as other vehicles of intelligence on the Continent of Europe, having ftated, that inflammations of the arm had continued obftinate *in spite* of warm fomentations, I beg leave to exprefs my humble opinion, that they continued obftinate *in consequence* of thofe warm fomentations; and I hope I fhall be excufed for expreffing alfo my furprife, that, in an age when the means of information are fo acceffible, and when medical fcience is fo far improved, one of its moft incontrovertible axioms, that heat acts as a ftimulus, and aggravates inflammation, can be fo totally forgotten. I am, &c.

JOHN RING.

*New Street, Hanover Square, Nov. 17, 1801.*

